# Supplementary material for: Variations in Human Milk Metabolites After Gestational Diabetes: Associations with Infant Growth
Source: Nutrients. 2025 Apr 26;17(9):1466. doi: 10.3390/nu17091466 (PMC12073254; doi:10.3390/nu17091466)
Supplement: Supplementary file 1 [file nutrients-17-01466-s001.zip › nutrients-3590165-supplementary.pdf]

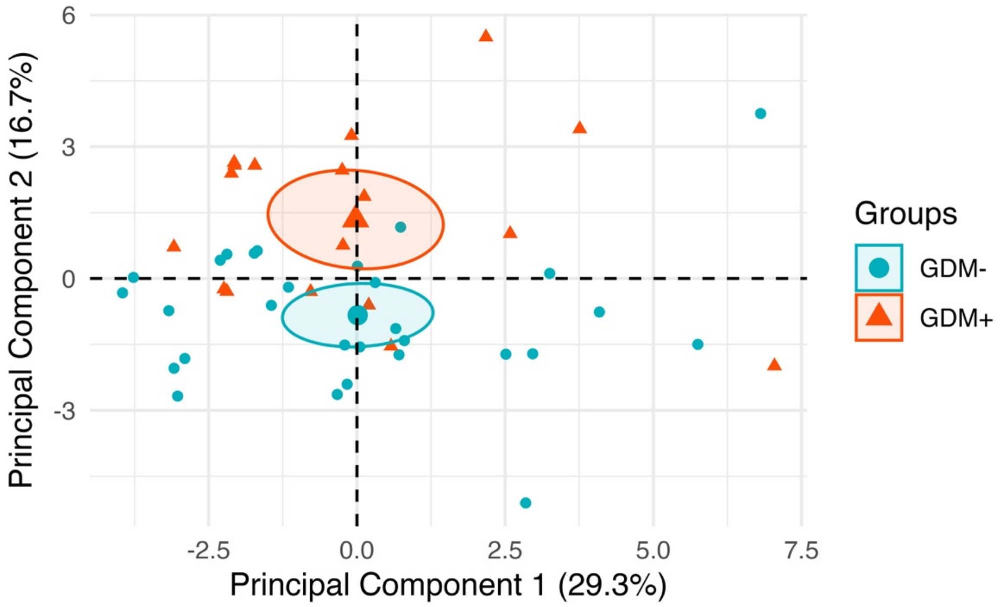

Figure S1: Visualization of the Principal component analysis (PCA) modelling the human milk metabolite profile, excluding the two participants in the GDM+ group who had not exclusively breastfed

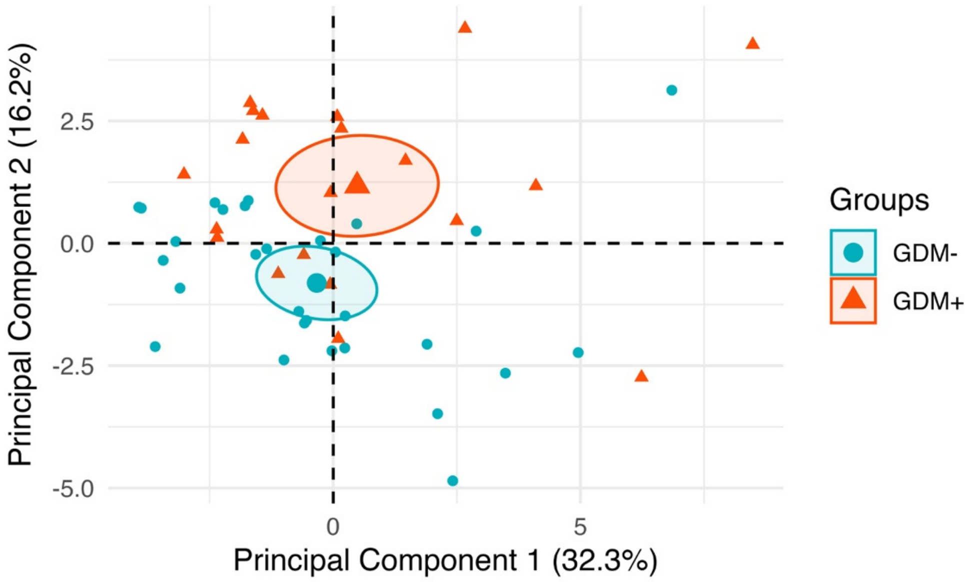

Figure S2: Visualization of the Principal component analysis (PCA) modelling the human milk metabolite profile, adjusting for fat mass percentage

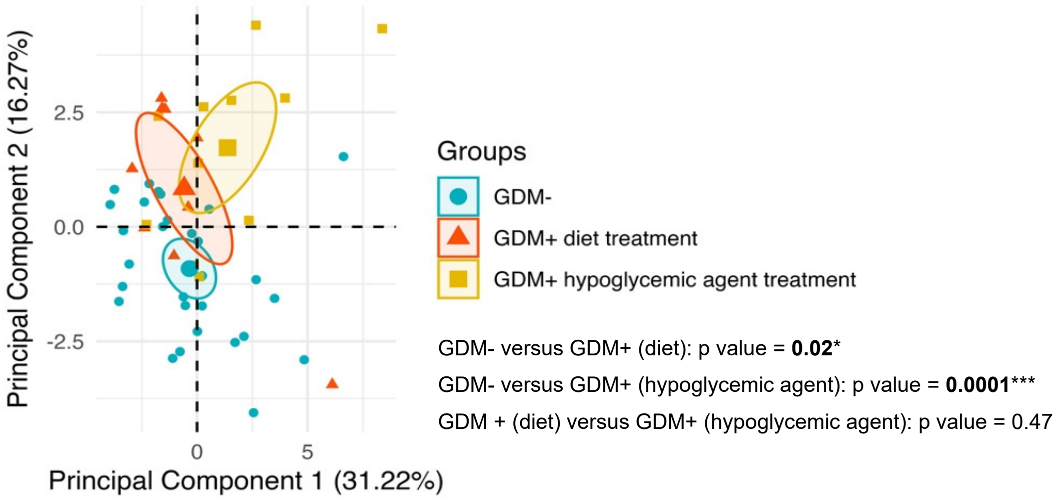

Figure S3: Visualization of the Principal component analysis (PCA) modelling the human milk metabolite profile, according to the treatment. * *p* value < 0.05, and *** *p* value < 0.001.

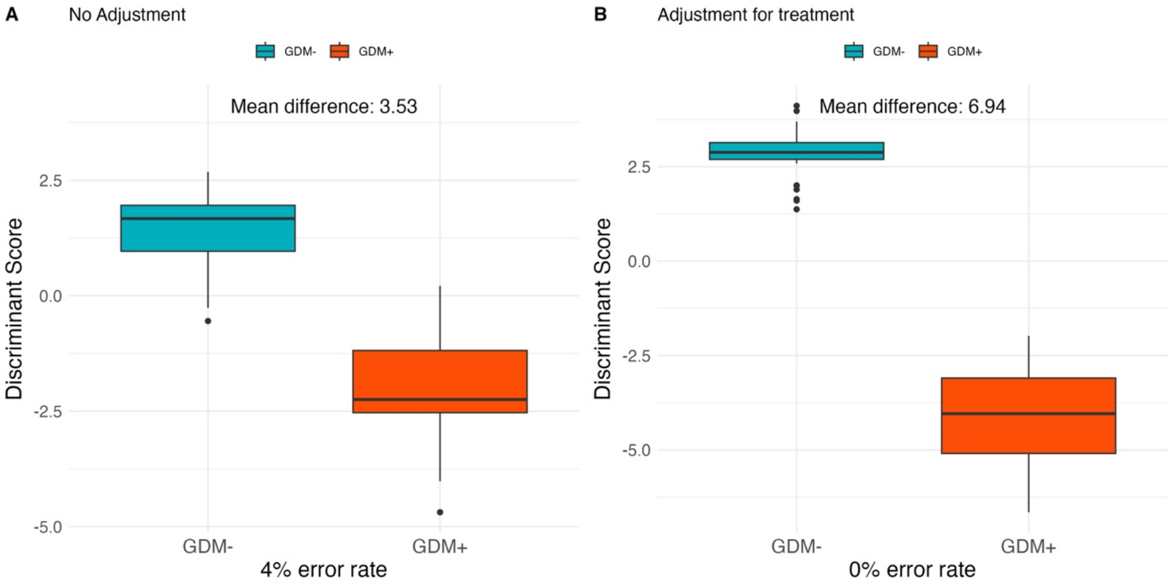

Figure S4: Linear Discriminant analysis (LDA) modeling the impact of treatment adjustment on metabolite profiles by GDM status, with error rate of the model.
